# LIFR-Mediated ERBB2 Signaling Is Essential for Successful Embryo Implantation in Mice

**DOI:** 10.3390/biom15050698

**Published:** 2025-05-10

**Authors:** Jumpei Terakawa, Sakura Nakamura, Mana Ohtomo, Saki Uehara, Yui Kawata, Shunsuke Takarabe, Hibiki Sugita, Takafumi Namiki, Atsuko Kageyama, Michiko Noguchi, Hironobu Murakami, Naomi Kashiwazaki, Junya Ito

**Affiliations:** 1 Graduate School of Veterinary Science, Azabu University, Kanagawa 252-5201, Japanh-murakami@azabu-u.ac.jp (H.M.);; 2 Laboratory of Toxicology, School of Veterinary Medicine, Azabu University, Kanagawa 252-5201, Japan; 3 Laboratory of Animal Reproduction, School of Veterinary Medicine, Azabu University, Kanagawa 252-5201, Japan; 4 Laboratory of Theriogenology, School of Veterinary Medicine, Azabu University, Kanagawa 252-5201, Japan; 5 Laboratory of Infectious Diseases, School of Veterinary Medicine, Azabu University, Kanagawa 252-5201, Japan

**Keywords:** embryo implantation, LIF, LIFR, Gp130, ERBB2

## Abstract

In eutherian mammals, embryo implantation is a critical process for a successful pregnancy. In mice, the activation of the leukemia inhibitory factor (LIF) receptor–STAT3 signaling axis induces embryo adhesion and decidualization. The LIF receptor is believed to function as a heterodimer composed of LIFR (encoded by *Lifr*) and GP130 (encoded by *Il6st*); however, their distinct expression patterns in the uterine epithelium immediately prior to implantation suggest divergent functional roles. In this study, we generated uterine epithelium-specific *Lifr* knockout (*Lifr* eKO) mice and conducted a comprehensive gene expression analysis of the endometrium before implantation. We compared these results with those from uterine epithelium-specific *Gp130* knockout (*Gp130* eKO) mice. Similarly to *Gp130* eKO mice, *Lifr* eKO mice were completely infertile. We identified 299 genes with expression changes greater than twofold following gene deletion; among these, 31 genes were downregulated and 57 genes were upregulated in both eKO models. Many of the downregulated genes were previously implicated in uterine function. Hub gene analysis identified *Erbb2* and *c-Fos* as key regulators in both models. Further experiments using an ERBB2 inhibitor suggested that LIFR–ERBB2-mediated signaling plays a crucial role in embryo implantation.

## 1. Introduction

Infertility, defined as the inability to conceive after 12 months or more of regular unprotected sexual intercourse, affects millions of individuals worldwide [1]. It is estimated that approximately one in six individuals of reproductive age will experience infertility during their lifetime. Infertility can result from various factors affecting either the male or female reproductive system. Although the development of assisted reproductive technologies has facilitated infertility treatment, expanding access to these treatments is crucial for improving pregnancy rates and creating an optimal environment for conception [2]. In humans, the endometrium remains refractory to embryo implantation throughout most of the menstrual cycle, except during a brief period known as the implantation window. This window is estimated to occur 7 ± 2 days after ovulation, with the day of ovulation designated as day 0 [3,4]. The implantation window is tightly regulated by two key hormones, progesterone (P4) and 17β-estradiol (E2), and their effects have been extensively studied using human tissues, cultured cells, and animal models [5,6].

Elucidating the mechanisms underlying embryo implantation—the first point of interaction between the mother and the fetus—studies using genetically modified mouse models have been invaluable, leading to the identification of numerous key regulatory factors [6]. In both humans and mice, embryo implantation involves attachment, adhesion, and the invasion of the hatched blastocyst into the receptive endometrium, accompanied by decidualization [7]. Similarly to humans, mice exhibit an implantation window regulated by P4 and E2 [6]. In mice, following mating, the presence of a vaginal plug is designated as gestation day 1 (D1). A transient surge in E2 on D4, in the presence of P4 secreted by the corpus luteum formed after ovulation, establishes the implantation window [8]. In ovariectomized mice, continuous P4 administration allows the embryo to remain in a hatched blastocyst state within the uterus for up to two weeks (delayed implantation). A single E2 injection triggers implantation and decidualization. In 1992, Stewart et al. demonstrated that female mice lacking the leukemia inhibitory factor (*Lif*) gene were infertile [9]. In mice, endometrial *Lif* expression is induced by nidatory E2, and the administration of the recombinant LIF protein rescues implantation in *Lif*-deficient mice, highlighting LIF as a crucial regulator of embryo implantation and decidualization [10]. LIF, a member of the interleukin-6 (IL-6) cytokine family, has multiple functions, including the maintenance of embryonic stem cell pluripotency [11]. IL-6 family cytokines share the GP130 (interleukin-6 signal transducer, encoded by *Il6st*) receptor subunit. LIF signals through a heterodimeric receptor complex composed of the GP130 and LIF receptor (LIFR), leading to the activation of the Janus kinase (JAK)–signal transducer and activator of the transcription 3 (STAT3) signaling pathway [12,13]. In addition to LIF, other IL-6 family cytokines, such as oncostatin M (OSM) and cardiotrophin-1 (CT-1), also bind to the GP130/LIFR complex. Notably, CT-1 administration can induce implantation in delayed-implantation mouse models, similar to LIF, underscoring the critical role of GP130/LIFR signaling in embryo implantation [14].

GP130/LIFR signaling plays a pivotal role in the decidualization of endometrial stromal cells, supporting embryonic invasion and placentation in mice. The significance of GP130/LIFR signaling in the uterine epithelium has been demonstrated through studies using genetically engineered mouse models with uterine epithelium-specific gene deletions. Female mice lacking *Stat3* (*Stat3* eKO) [15], *Gp130* (*Gp130* eKO) [16], or *Lifr* (*Lifr* eKO) [17,18] exhibit infertility due to implantation failure. Our previous studies showed that *Gp130* eKO mice display defective epithelial remodeling, reduced hormone responsiveness, and immune cell infiltration in the endometrium [16]. Although GP130 and LIFR function as heterodimeric receptors, their distinct expression patterns in the endometrium during implantation suggest potential differences in their roles. To further elucidate the commonalities and specificities of GP130 and LIFR signaling in embryo implantation, we analyzed *Lifr* eKO mice using the comprehensive gene expression profiling of preimplantation uterine tissues. This study identifies the essential signaling pathways involved in embryo implantation and decidualization, providing novel insights into the molecular mechanisms underlying infertility.

## 2. Materials and Methods

### 2.1. Animals

All animal procedures were approved by the Ethical Committee for Vertebrate Experiments at Azabu University (ID#200312-24). All experiments were conducted in accordance with the relevant guidelines and regulations, including the Animal Research: Reporting of In Vivo Experiments (ARRIVE) guidelines. The following mouse strains, all aged over seven-week-old, were used in the experiments: *Ltf^iCre/+^* mouse <*Ltf^tm1(icre)Tdku^/J*, JAX: 026030> [19], and *Lifr^tm1a(EUCOMM)Hmgu^* (strain ID: EM:06941) mouse purchased from the European Mouse Mutant Archive (EMMA). To obtain the *Lifr^flox/flox^* mice, *Lifr^tm1a(EUCOMM)Hmgu^* mice were crossed with FLPe transgenic mice <Tg(CAG-flpe)36Ito> (RBRC01834) [20]. FRT-LacZ-neo-FRT cassette was removed, and loxP-flanked exon 4 of the *Lifr* gene was left in *Lifr^flox/flox^* mice. *Lifr^flox/flox^* mice were then crossed with *Ltf^iCre/+^* mice. *Lifr^flox/flox^* mice harboring *Ltf^iCre^* were used as the uterine epithelial conditional knockout of *Lifr* (*Lifr* eKO), and *Lifr^flox/flox^* mice were used as the control. The primers used for genotyping were as follows: 5′-GTTTCCTCCTTCTGGGCTCC-3′, 5′-TTTAGTGCCCAGCTTCCCAG-3′ and 5′-CCTGTTGTTCAGCTTGCACC-3′ for *Ltf^iCre^*; 5′-TGAGAGCACGGAAGCTCTTT-3′ and 5′-ACTGCCCGACAAGGTTTTTA-3′ for *Lifr^flox^*. All strains were maintained on the C57BL/6J background purchased from Jackson Laboratory Japan (Kanagawa, Japan) and housed in the barrier facility at Azabu University. All mice were fed ad libitum under controlled light–dark cycles (12 h light/12 h dark) at 23 ± 2 °C. The first day of pregnancy (D1) was designated as the morning when a vaginal plug was observed in the female that had been mated with fertile wild-type males on the previous evening. The implantation sites were visualized by an intravenous injection of 0.1 mL/head of 1% Chicago sky blue 6B (C8679, Sigma-Aldrich, St. Louis, MO, USA) dissolved in saline, as previously described [16]. Artificial decidualization was performed by intraluminal injections of 0.02 mL sesame oil (S3547, Sigma-Aldrich) into one side of the uterine hone after females were mated with a vasectomized male as previously described [16]. Mice were euthanatized by cervical dislocation after receiving a combination anesthetic with 0.75 mg/kg of medetomidine (Meiji Animal Health, Kumamoto, Japan), 4.0 mg/kg of midazolam (Sandoz, Tokyo, Japan), and 5.0 mg/kg of butorphanol (Meiji Animal Health). Uterine tissues were dissected and fixed with 4% paraformaldehyde (PFA) solution in phosphate-buffered saline (PBS) for histological study and snap-frozen and kept at −80 °C until they were used for RNA extraction.

For drug treatment, tucatinib (1 mg/400 μL/ head, T2346, TargetMol, Boston, MA, USA) or sapitinib (1 mg/400 μL/ head, AZD8931, Selleck Chemicals, Houston, TX, USA), dissolved in 10% dimethyl sulfoxide (D2650, Sigma-Aldrich), 0.5% methylcellulose (M0262, Sigma-Aldrich), and 0.1% polysorbate 80 (P8074, Sigma-Aldrich) in distilled water, was orally administered to female mice on D3 (1800–1900 h) and D4 (0900–1000 h). The uterus was rinsed with saline if no blue band was found after blue dye injection on D5. If no blastocyst was recovered, it was excluded from the analysis.

### 2.2. Histological Analysis

**Figure 1:**
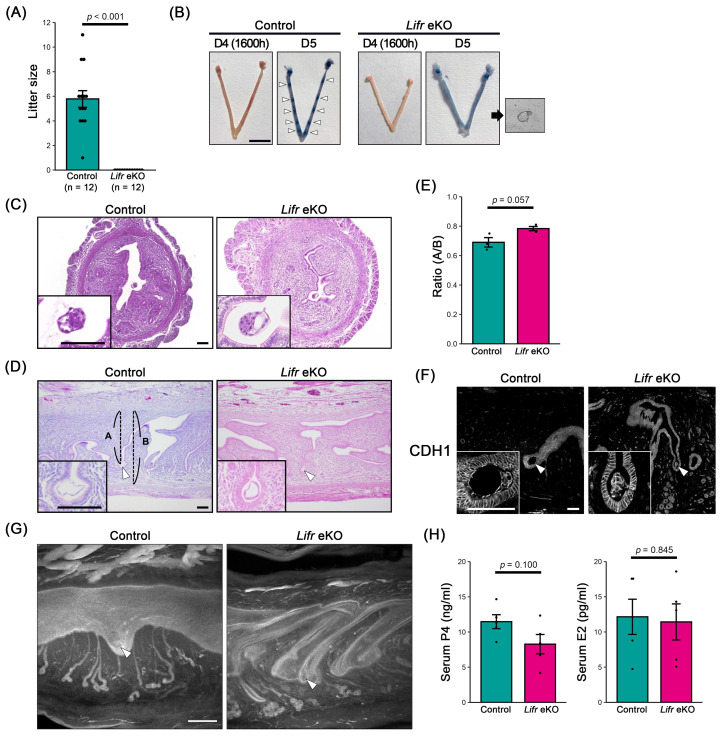
Uterine epithelium-specific deletion of *Lifr* (*Lifr* eKO) results in female infertility. (**A**) Pregnancy outcomes in control and uterine epithelium-specific *Lifr* (*Lifr* eKO) females mated to wild-type males. (**B**) Representative gross morphology of uteri from control and *Lifr* eKO females on D4 (1600 h) and D5. Implantation sites (indicated by white arrowheads) are visualized through blue dye injection on D5. Hatched blastocysts (shown in the inset) were recovered from the *Lifr* eKO uterus. Scale bar = 1 cm. (**C**) Cross-sectional images of uteri on D4 (1600 h). Scale bar = 100 µm. (**D**) Longitudinal sections of uteri on D5. Scale bar = 100 µm. Arrowheads indicate blastocysts. “A” indicates the distance from the mesometrial edge to the center of the blastocyst. “B” indicates the distance from the mesometrial edge to the anti-mesometrial edge. (**E**) The depth of the uterine chamber (A/B ratio shown in panel D) is comparable between the control and *Lifr* eKO females. (**F**) Immunostaining for CDH1 on D5. Scale bar = 100 µm. Arrowheads indicate blastocysts. (**G**) Whole-mount staining for CDH1 on D5. Scale bar = 200 µm. Arrowheads indicate blastocysts. (**H**) Serum levels of progesterone (P4) and 17β-estradiol (E2) on D4.

Fixed uterine tissues were paraffin-embedded, and paraffin sections (6 µm) were stained with hematoxylin and eosin (H&E). The embryo position in the uterine lumen on D5 was determined by dividing the distance from the mesometrial edge to the center of the embryo (referred to as “A” in Figure 1D) by the distance from the mesometrial edge to the anti-mesometrial edge (referred to as “B” in Figure 1D) as described [16].

### 2.3. Immunostaining

Immunostaining was performed as previously described [16]. Briefly, the paraffin sections (6 µm) were deparaffinized, hydrated, and used for antigen retrieval by autoclaving in 10 mM sodium citrate buffer (pH = 6.0) for 5 min. For immunohistochemistry (IHC), the sections were further incubated in 3% hydrogen peroxide diluted with methanol for 15 min. After blocking with non-specific staining blocking reagent (X0909, Dako, Carpinteria, CA, USA), the slides were incubated with primary antibodies (shown in Table 1) overnight at 4 °C. The same slides were subjected to incubation with the Histofine mouse stain kit (Nichirei Biosciences, Tokyo, Japan) for 1 h. Signals were visualized by 3,3′-diaminobenzidine tetrahydrochloride (DAB) and counterstained with hematoxylin.

For the immunofluorescence (IF) evaluation, the slides were incubated with Alexa Fluor 488-conjugated secondary antibodies (Jackson Immuno Research Laboratories, West Grove, PA, USA) for 1 h and mounted with ProLong Glass Antifade Mountant with NucBlue Stain (P36981, Thermo Fisher Scientific, Waltham, MA, USA). Micrographs were captured by PROVIS AX80 microscopy (Olympus, Tokyo, Japan) or BZ-X700 microscopy (Keyence, Osaka, Japan). All signals were detected under the same lighting conditions for the control and *Lifr* eKO groups.

### 2.4. Whole-Mount Staining

The whole-mount staining of the uterine horn was performed as previously described with some modifications [21]. In brief, uteri were fixed in a mixture of dimethyl sulfoxide (DMSO, D5879, Sigma-Aldrich)/methanol (1:4), followed by methanol/PBS containing 1% Triton-X100 (Sigma-Aldrich) (1:1) for 15 min at room temperature (RT), blocked by PBS containing 1% Triton-X100 and 2% skim milk (198-10605, Fujifilm Wako Pure Chemical, Osaka, Japan) for 2 h at RT and incubated with a primary antibody for CDH1 (#3195, Cell signaling technology, Danvers, MA, USA); they were then diluted in a blocking buffer for 5 nights at 4 °C. Uteri were washed with the blocking buffer six times for 30 min each, and then incubated with a secondary antibody diluted in a blocking buffer for 2 nights at 4 °C. Uteri were washed with the blocking buffer six times for 30 min each, and incubated in methanol for 30 min and methanol containing 3% H_2_O_2_ overnight. Uteri were washed in methanol and cleaned overnight using BABB (benzyl alcohol/benzyl benzoate, 1:2; benzyl alcohol from Kanto Chemical, Tokyo, Japan; benzyl benzoate from Fujifilm Wako). Images were captured by the Leica TCS SP5 II confocal microscope (Wetzlar, Germany).

### 2.5. Measurement of Serum Hormone Levels

Blood samples from control and *Lifr* eKO mice were collected on D4. The serum was separated by centrifugation (4 °C, 6000× *g*, 7 min) and stored at −80 °C until analysis. The serum concentrations of progesterone (P4) and 17β-estradiol (E2) were measured by the enzyme-linked immunosorbent assay as described previously [22].

### 2.6. RNA Extraction and RNA Sequence (RNA-Seq)

Total RNA was isolated using the RNeasy plus mini kit (Qiagen, Hilden, Germany) according to the manufacturer’s instructions. RNA-seq was carried out by Azenta Life Sciences (Burlington, MA, USA). Briefly, 1 μg of total RNA was used following library preparation. The poly(A) mRNA isolation was performed using Oligo(dT) beads. mRNA fragmentation was performed using divalent cations at a high temperature. Priming was performed using Random Primers. First-strand cDNA and second-strand cDNA were synthesized. Purified double-stranded cDNA was then treated to repair both ends and add dA-tailing in one reaction, followed by a T-A ligation to add adaptors to both ends. The size selection of adaptor-ligated DNA was then performed using DNA Clean Beads. Each sample was then amplified by PCR using P5 and P7 primers, and the PCR products were validated. Then, libraries with different indices were multiplexed and loaded on an MGI2000 instrument for sequencing using a 2 × 150 paired-end (PE) configuration according to the manufacturer’s instructions. The FASTQ files were processed for the removal of adaptor sequences, trimmed, and quality-based filtered using fastp v0.23.2 [23]. The trimmed reads had ribosomal RNA removed using SortMeRNA v4.3.6 [24]. The removed reads were mapped onto the reference genome of Mus musculus (GRCm39) using STAR v2.7.10a [25]. Mapped reads were counted using RSEM v1.3.3 [26]. Differential expression analysis was performed in R v4.2.1 (URL https://www.R-project.org/ accessed on 11 January 2022) using the EdgeR v 3.38.4 package [27], and significantly different genes were visualized using an MA plot and heatmap using the gplots v3.1.3 and genefilter v1.78.0 packages. A volcano plot was generated using R with the EnhancedVolcano package (v1.22.0). Gene ontology analysis was performed by ShinyGO 0.77 [28] with DEGs as the input. A Venn diagram was drawn using (https://bioinformatics.psb.ugent.be/webtools/Venn/ accessed on 14 March 2025).

### 2.7. Statistical Analysis

All of the data are presented as the mean ± standard error of the mean (SEM). Differences in the data were examined with the use of Student’s *t*-test or the Mann–Whitney *U*-test. A *p*-value < 0.05 was considered significant. Embryo implantation rates after ERBB2 inhibitor treatment were tested using the chi-square test.

### 2.8. Data Availability

The datasets generated and/or analyzed during the current study are available in the GEO repository, accession number GSE293425.

https://www.ncbi.nlm.nih.gov/geo/query/acc.cgi?acc=GSE293425 (accessed on 7 May 2025).

## 3. Results

### 3.1. Epithelial-Specific Lifr-Deficient Females Are Infertile

To assess whether uterine epithelium-specific *Lifr* knockout (*Lifr* eKO) females exhibit infertility, we examined their reproductive phenotype. All control females gave birth to an average of 6 ± 1 pups per litter (*n* = 12), whereas *Lifr* eKO females did not produce any offspring, indicating complete infertility (*n* = 12) (Figure 1A). To determine whether implantation failure was responsible for this phenotype, we performed blue dye injection assays on day 5 of gestation (D5) to visualize implantation sites. In control females, distinct blue bands indicating successful implantation were observed. In contrast, no implantation sites were detected in *Lifr* eKO females, and hatched blastocysts were recovered from the uterine lumen (Figure 1B). These results confirm that infertility in *Lifr* eKO mice is due to implantation failure, with no evidence of decidualization.

Hematoxylin and eosin (HE) staining of D4 uteri revealed that blastocysts were properly positioned on the anti-mesometrial side of the slit-shaped uterine lumen in both control and *Lifr* eKO females (Figure 1C). Further analysis of D5 uterine sections confirmed that blastocysts in *Lifr* eKO mice remained on the anti-mesometrial side, similar to controls (Figure 1D,E), and were surrounded by the uterine epithelium (Figure 1F). Whole-mount uterine staining using the epithelial marker CDH1 demonstrated that, in control females, morphological changes associated with decidualization occurred around the blastocyst upon implantation. However, in *Lifr* eKO females, the slit-like uterine lumen remained unchanged, indicating a lack of decidual response (Figure 1G).

To determine whether hormonal abnormalities contributed to implantation failure, we measured serum levels of P4 and E2 on D4. No significant differences were observed between the control and *Lifr* eKO females (Figure 1H), suggesting that implantation failure in *Lifr* eKO mice is not due to hormonal insufficiency.

### 3.2. No Decidual Reaction in Epithelial-Specific Lifr-Deficient Females

**Figure 2:**
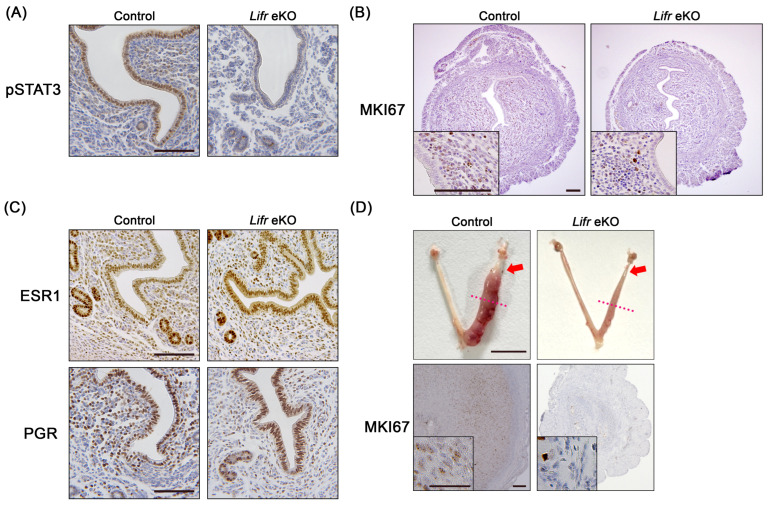
Histochemical characterization in the Lifr eKO uterus prior to embryo implantation. (**A**) Immunostaining for phosphorylated STAT3 (pSTAT3) on D4 (1600 h). Scale bar = 100 µm. (**B**) Cell proliferation status assessed by MKI67 immunostaining on D4 (1600 h), with the inset showing higher magnification. Scale bar = 100 µm. (**C**) The expression of estrogen receptor alpha (ESR1) and progesterone receptor (PGR) on D4 (1600 h). Scale bar = 100 µm. (**D**) The representative gross morphology of uteri after the artificial decidualization of stimuli in control and Lifr eKO mice (upper panels). Scale bar = 1 cm. Red arrows indicate the site of sesame oil injection, and red dashed lines mark the point of sectioning. Immunostaining for MKI67 in uteri (lower panels). Scale bar = 200 µm; inset scale bar = 50 µm.

LIF secreted during implantation induces the heterodimerization of LIFR and GP130 in the uterine epithelium, leading to the phosphorylation of STAT3 by Janus tyrosine kinase (JAK) and the subsequent transcriptional activation of target genes [29]. Since *Lifr* eKO mice lack LIFR in the uterine epithelium, only a few cells exhibited phosphorylated STAT3 (pSTAT3) on D4 (16:00 h) in the luminal epithelium of *Lifr* eKO mice in this study (Figure 2A). The persistence or cessation of uterine epithelial cell proliferation is closely linked to the success or failure of embryo implantation [30,31]. In *Lifr* eKO females, epithelial cell proliferation ceased on D4 (16:00 h), similar to the controls (Figure 2B), which is consistent with previous findings [17]. An analysis of steroid hormone receptor expression revealed no significant differences in the levels of estrogen receptor 1 (ESR1) and the progesterone receptor (PGR) on D4 between *Lifr* eKO and control mice (Figure 2C). However, *Lifr* eKO females failed to undergo a decidualization response upon implantation. Furthermore, artificial decidualization induced by oil injection into the uterine lumen elicited no response in *Lifr* eKO mice, as evidenced by the absence of stromal cell differentiation and proliferation (Figure 2D).

### 3.3. Gene Expression Profiles in the Uterus of Epithelial-Specific Lifr-Deficient Mice During Embryo Implantation

**Figure 3:**
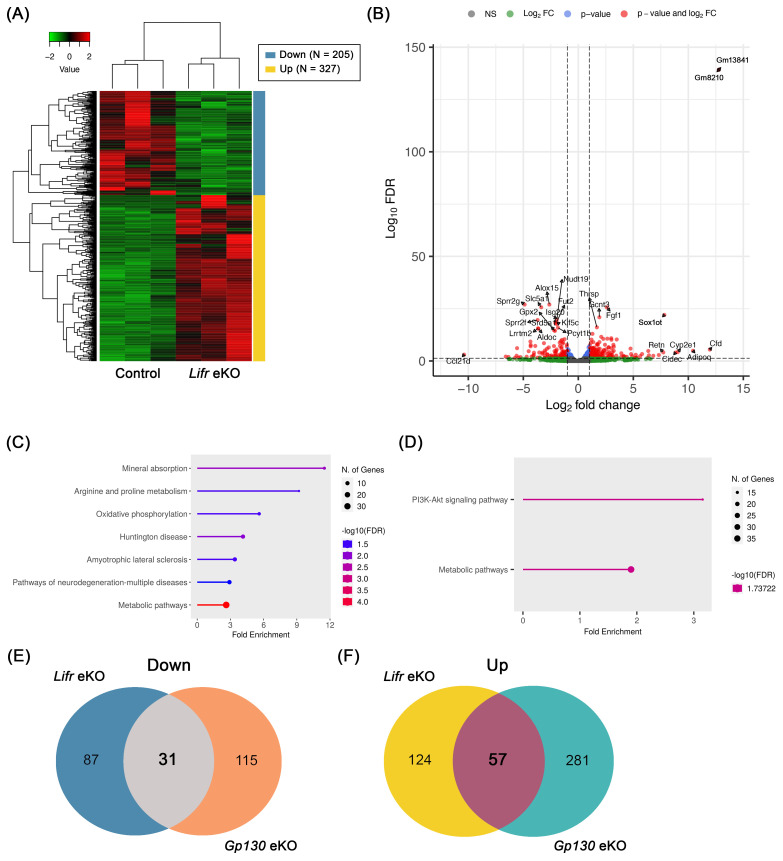
Comprehensive gene expression analysis between control and *Lifr* eKO uterus before embryo implantation. (**A**) Heatmap displaying genes with altered expression levels in *Lifr* eKO uteri (FDR < 0.05). (**B**) Volcano plot showing differentially expressed genes (DEGs) that are either downregulated or upregulated in *Lifr* eKO compared to controls. (**C,D**) Gene ontology (GO) analysis of downregulated (**C**) and upregulated (**D**) DEGs in *Lifr* eKO uteri compared to controls, highlighting systematic characteristics of these genes. (**E,F**) Venn diagrams illustrating the overlap of downregulated (**E**) and upregulated (**F**) DEGs between *Lifr* eKO and *Gp130* eKO uteri.

To further investigate the molecular mechanisms underlying infertility in *Lifr* eKO mice, we performed RNA sequencing (RNA-seq) on preimplantation uterine tissues from *Lifr* eKO and control mice. A total of 532 differentially expressed genes (DEGs) were identified based on a false discovery rate (FDR) threshold of <0.05, comprising 205 downregulated and 327 upregulated genes in *Lifr* eKO compared to the controls (Figure 3A, Supplemental Data S1). Among these, 118 genes exhibited at least a twofold decrease in expression, while 181 genes showed a twofold increase (Figure 3B). Functional enrichment analysis of the downregulated genes revealed their association with various cellular processes, including mineral absorption, arginine and proline metabolism, oxidative phosphorylation, and metabolic pathways (Figure 3C). In contrast, upregulated genes were primarily linked to the PI3K-Akt signaling pathway and other metabolic pathways (Figure 3D). Given that we previously performed RNA-seq analysis on *Gp130* eKO uteri [16], we examined the overlap between the DEGs identified in *Lifr* eKO and *Gp130* eKO models. We found that 31 genes were commonly downregulated, while 57 genes were commonly upregulated more than twofold in both knockout models (Figure 3E,F, Supplemental Data S2).

The further hub gene analysis of DEGs in *Lifr* eKO uteri identified *Erbb2* as a key regulator. *Erbb2* is linked to several factors previously implicated in embryo implantation, including *Hif1a*, *Fgfr2*, *Areg*, *Msx1*, and *Fgf1*. Additionally, *Fos* (*c-fos*), previously identified as a hub gene in *Gp130* eKO uteri, was also highlighted in the present analysis. These findings suggest that a gene network centered on ERBB2 may play a crucial role in embryo implantation and decidualization.

### 3.4. The Role of Erbb2 in Embryo Implantation

**Figure 4:**
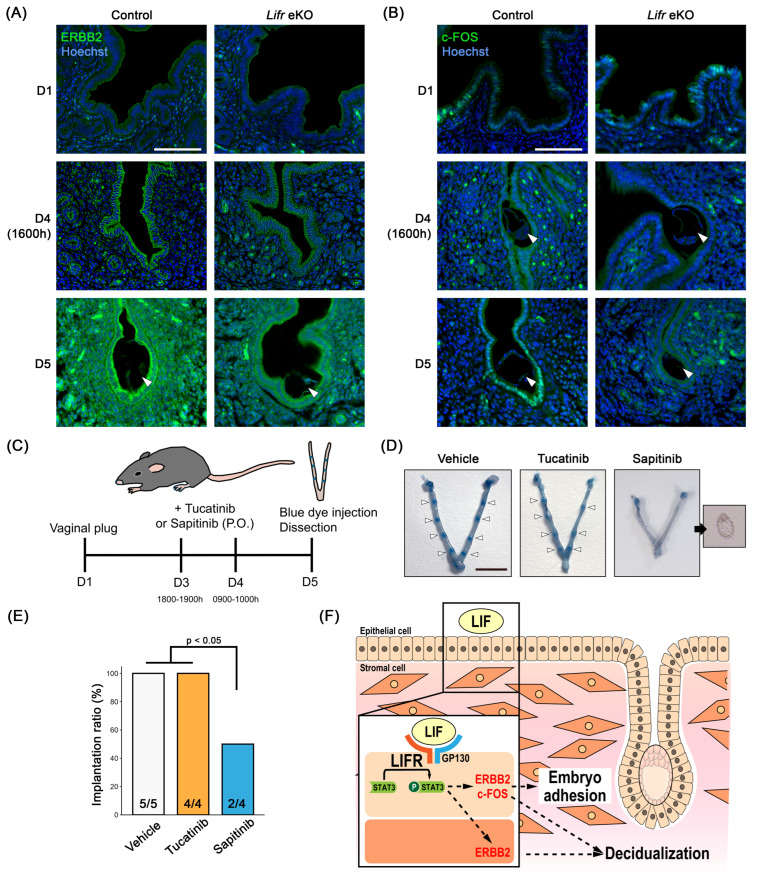
ERBB2 signaling is essential for embryo implantation. (**A,B**) Immunostaining for ERBB2 (**A**) and c-FOS (**B**) in the pregnant uterus. Scale bar = 100 µm. Arrowheads indicate blastocysts. (**C**) The experimental procedure for administering ERBB2 inhibitors to pregnant females. (**D**) Representative images of gross uterine morphology from control and drug-treated mice. Arrowheads indicate implantation sites. Scale bar = 1 cm. (**E**) Sapitinib treatment inhibits up to 50% of embryo implantation. (**F**) A model illustrating LIFR-mediated embryo implantation in mice.

ERBB2 expression was low on D1 but was detected in the uterine epithelium prior to embryo implantation. Following implantation, its expression increased in the luminal epithelium and the primary decidual zone (Figure 4A). In contrast, ERBB2 expression was weak in *Lifr* eKO uteri, where embryo implantation failed to occur. *c-Fos* expression was predominantly observed in epithelial cells in both control and *Lifr* eKO mice on D1 (Figure 4B). In control mice, a marked increase in *c-Fos* expression was detected from D4 to D5, whereas no such increase was observed in *Lifr* eKO mice. To investigate whether *Erbb2* signaling is directly involved in embryo implantation, we administered two ERBB2 inhibitors, tucatinib, and sapitinib, orally before implantation (Figure 4C). Blue dye injections revealed implantation sites in 100% of the cases treated with tucatinib, similar to the control group. However, in 50% of sapitinib-treated mice, no implantation sites were detected, and blastocysts were recovered instead (Figure 4D,E). These results suggest that the partial inhibition of ERBB2 signaling can lead to implantation failure, highlighting its potential role in successful embryo implantation.

## 4. Discussion

The present study confirms that the uterine epithelium-specific deletion of the *Lifr* gene leads to complete infertility. This finding is consistent with a previous report by Fukui et al., who analyzed the phenotype using the same *Ltf-Cre* knock-in strain [17]. In contrast, Cheng et al. also reported uterine epithelium-specific *Lifr* deletion; however, incomplete gene deletion in their study resulted in implantation sites in fewer than 40% of the individuals, likely due to the use of a different *Ltf-Cre* knock-in mouse strain [18]. In the present study, *Lifr* eKO mice exhibited complete implantation failure, with no decidual response observed. Importantly, at the preimplantation stage (D4, 1600 h), the *Lifr* eKO uterus was comparable to that of the control group in terms of serum levels of sex steroid hormones, the expression of their receptors, and blastocyst positioning on the anti-mesometrial side of the uterine lumen. However, the absence of STAT3 phosphorylation in the uterine epithelium at the same time point suggests that the presence or absence of activated STAT3 plays a critical role in determining subsequent implantation outcomes. Indeed, in agreement with the findings of Fukui et al., by day 5 of gestation, control females exhibited crypt-like structures at the implantation sites, whereas no such morphological changes were observed in *Lifr* eKO mice.

In this study, we conducted a comprehensive gene expression analysis of the *Lifr* eKO uterus on D4 (1600 h) and compared the results with our previous analysis of *Gp130* eKO [16]. We identified 299 differentially expressed genes (*Lifr* eKO: 118 downregulated and 181 upregulated) compared to 484 genes in the *Gp130* eKO dataset (146 downregulated and 338 upregulated). Notably, the PI3K-Akt signaling pathway, which plays a crucial role in E2-dependent uterine cell proliferation [32], was altered in both models. While the inhibition of epithelial cell proliferation—a prerequisite for successful embryo implantation [33]—was observed in both *Lifr* eKO and *Gp130* eKO, disruptions in cell signaling pathways may also contribute to the observed implantation failure. A total of 31 genes were commonly downregulated in both *Lifr* and *Gp130* eKO models. Many of these genes are associated with uterine function, including *Alox15*, which plays a role in lipid metabolism and inflammatory responses and is markedly downregulated in the *Gp130* eKO uterine epithelium [16]. *Coch* has been identified as an early *Lif* target gene, although its function remains unclear due to the absence of an overt phenotype in null mutants [34]. Several genes implicated in preimplantation processes were also downregulated, such as *Slc46a2* and *Cyp26a1* [35], *Prss23* [36], *Pstpip2*—which is highly expressed in the epithelium surrounding the implantation site [37]—and *Tm4sf4*, which is a candidate factor for embryo attachment in humans [38]. Additionally, multiple genes affected by sex steroid hormones were dysregulated, including *Trim15* [39] and *Entpd3* [40], as well as epithelial-specific factors such as *Sprr2a2* [41] and *Lgr5* [42]. The anion exchanger *Slc26a6*, which plays a key role in uterine lumen pH regulation [43], was also downregulated. *Mcub*, involved in mitochondrial remodeling during stromal cell differentiation [37], and *Srd5a1*, which facilitates decidualization by converting testosterone into the potent androgen 5α-dihydrotestosterone (DHT) [44], were among the affected genes. Furthermore, *Slc22a4* [44] and *Gpx2* [45]—both linked to uterine gland development—were also differentially expressed.

In this study, we also identified hub genes associated with expression variation: *Erbb2* in *Lifr* eKO and *Fos* (*c-fos*) in *Gp130* eKO. A previous study by Kim et al. demonstrated that heparin-binding EGF (HB-EGF), which is essential for uterine epithelial–blastocyst crosstalk, regulates crypt-like space formation via the tyrosine phosphorylation of VANGL2 through the ERBB2/3 pathway [46]. These findings suggest that ERBB2-mediated signaling plays a crucial role in the establishment of embryo implantation. Interestingly, uterine-specific *Erbb2*-deficient mice exhibited a significant reduction in litter size but did not show complete infertility, whereas epithelial-specific *Erbb2* deletion had no effect on fertility. However, the implantation phenotype was more severe in mice who were deficient in both uterine-specific *Erbb2* and *Erbb3*. This suggests functional redundancy between these receptors. The differential effects of ERBB2 inhibition by small-molecule inhibitors further support this notion: while tucatinib, a potent ERBB2 inhibitor [47], did not prevent implantation, sapitinib, which suppresses ERBB2, the epidermal growth factor receptor (EGFR), and ERBB3 [48], leading to implantation failure. These results imply that ERBB3 compensates for ERBB2’s function in uterine epithelial signaling during implantation. Additionally, c-FOS, a nuclear phosphoprotein and transcription factor activated by E2 [49], was expressed in epithelial cells of both control and *Lifr* eKO uteri on day 1 of gestation. However, by D5, c-FOS expression was observed exclusively in the control epithelium surrounding the embryo, while it was absent in *Lifr* eKO. Given that the uterine epithelium in direct contact with the blastocyst undergoes collapse to facilitate embryonic invasion, these findings suggest that GP130/LIFR-STAT3-mediated signaling regulates epithelial remodeling via c-FOS expression, which is a process critical for successful implantation.

The loss of epithelial cell polarity, which is required for the acquisition of endometrial receptivity, is recognized in the human endometrium as plasma membrane transformation (PMT) [50]. In mice, epithelial cell polarity is also lost at the time of implantation, followed by trophoblast trophectoderm-mediated entosis, which disrupts the epithelial barrier and allows for interaction with the underlying stroma [51]. It has been shown that LIF signaling plays a critical role in regulating cell polarity through Msx1 and Wnt signaling pathways [52]. Both *Lifr* eKO and *Gp130* eKO mice exhibit complete failure of embryo adhesion, suggesting that the gene set identified in our study may be involved in regulating epithelial polarity. This possibility is further supported by previous findings, which identified that ERBB2 is involved in embryo implantation through VANGL2, a key component of planar cell polarity [46].

The critical role of GP130/LIFR-mediated signaling in the uterine epithelium for processes following blastocyst adhesion is underscored by the fact that female mice lacking uterine epithelium-specific *Stat3* [15], *Gp130* [16], or *Lifr* [17,18] all fail to support embryo adhesion. However, as previously reported by us and other research groups, the localization patterns of *Gp130* and *Lifr* during implantation differ significantly despite their presumed function as a heterodimer [16,53]. This suggests that there may be distinct functional roles for each receptor. To address these differences in detail, future studies should involve the analysis of uterine gland epithelial or luminal epithelial-specific gene deletions. For instance, gene deletions targeting the uterine glands can be induced using *Foxa2-Cre* [54] or *Prss29-Cre* [55]. However, it is important to note that FOXA2 is widely expressed in endoderm precursors and endoderm-derived organs, while *Prss29-Cre* has been shown to induce genetic defects after the first pregnancy. Genes highly expressed in the uterine glands, such as *Cxcl15* and *Rdh1*, may serve as potential candidates for the establishment of Cre transgenic or knock-in lines [56].

In humans, efforts have been made to identify markers for assessing endometrial receptivity; however, a comprehensive understanding of embryo–endometrium interactions has not yet been achieved [57]. Notably, the reduced expression of LIFR in the endometrial epithelium has been reported in women with unexplained infertility [58], suggesting that our findings may contribute to the identification of markers for endometrial receptivity in humans.

## 5. Conclusions

In this study, we identified the key factors involved in regulating embryo implantation by comparing *Lifr* eKO and *Gp130* eKO models (Figure 4F). Further analysis of the similarities and differences between the gene networks in these models is expected to provide a more comprehensive understanding of the underlying mechanisms.

## Figures and Tables

**Table 1:** Primary antibody list.

Target	Species	Source	Catalog No.	RRID	Dilution	Application
c-FOS	Rabbit	Abcam	ab222699	AB_2891049	1:1000	IF
ESR1	Rabbit	Abcam	ab32063	AB_732249	1:200	IHC
PGR	Rabbit	Abcam	ab101688	AB_10715248	1:200	IHC
MKI67	Rabbit	Abcam	ab16667	AB_302459	1:200	IHC
ERBB2	Mouse	Santa Cruz	sc-33684	AB_627996	1:200	IF
CDH1	Rabbit	Cell Signaling Technology	#3195	AB_2066683	1:200	IF
pSTAT3	Rabbit	Cell Signaling Technology	#9145	AB_2491009	1:200	IHC

## Data Availability

The datasets generated and/or analyzed during the current study are available in the GEO repository, accession number GSE293425. https://www.ncbi.nlm.nih.gov/geo/query/acc.cgi?acc=GSE293425 (accessed on 7 May 2025).

## Supplementary Materials

The following supporting information can be downloaded at https://www.mdpi.com/article/10.3390/biom15050698/s1. Data S1: The list of differentially expressed genes (DEGs) in *Lifr* eKO uteri compared with the control uteri on day 4 (1600 h) of pregnancy; Data S2: Down or upregulated DEGs in both *Lifr* eKO and *Gp130* eKO.

## Abbreviations

The following abbreviations are used in this manuscript:

P4	Progesterone
E2	17β-estradiol
LIF	Leukemia inhibitory factor
